# Schistosomiasis collection at NHM (SCAN)

**DOI:** 10.1186/1756-3305-5-185

**Published:** 2012-09-03

**Authors:** Aidan M Emery, Fiona E Allan, Muriel E Rabone, David Rollinson

**Affiliations:** 1 Wolfson Wellcome Laboratories, Dept of Life Sciences, Natural History Museum, Cromwell Road, London, SW7 5BD, UK

**Keywords:** *Schistosoma*, *Biomphalaria*, *Bulinus*, Biobank, Collection, Museum

## Abstract

**Background:**

The Natural History Museum (NHM) is developing a repository for schistosomiasis-related material, the Schistosomiasis Collection at NHM (SCAN) as part of its existing Wolfson Wellcome Biomedical Laboratory (WWBL). This is timely because a major research and evaluation effort to understand control and move towards elimination of schistosomiasis in Africa has been initiated by the Schistosomiasis Consortium for Operational Research and Evaluation (SCORE), resulting in the collection of many important biological samples, including larval schistosomes and snails. SCAN will collaborate with a number of research groups and control teams and the repository will acquire samples relevant to both immediate and future research interest. The samples collected through ongoing research and field activities, WWBL’s existing collections, and other acquisitions will be maintained over the long term and made available to the global research community for approved research purposes. Goals include: · Consolidation of the existing NHM schistosome and snail collections and transfer of specimens into suitable long-term storage systems for DNA retrieval, · Long-term and stable storage of specimens collected as part of on going field programmes initially in Africa especially relating to the SCORE research programmes, · Provision of access to snail and schistosome collections for approved research activities.

## Findings

### Background

The control of Neglected Tropical Diseases is not a trivial exercise; schistosomiasis alone is estimated to infect over 200 million people in more than 70 countries of the developing world, leading to chronic debilitating disease and up to 300,000 deaths per year [1]. The impact of disease both in terms of well-being and economy are truly colossal and this is especially true in sub-Saharan Africa where the vast majority of cases are found [2]. However, the landscape of schistosomiasis across the developing world is in a constant state of flux due to effective but often sporadic control programmes based on delivery of praziquantel and changing environmental pressures, either man made or climate induced [3-5].

To support field-based projects and provide specimens for retrospective study, the Natural History Museum (NHM) is expanding the capabilities of its Wolfson Wellcome Biomedical Laboratories (WWBL) to act as a global repository for schistosomiasis-related material. One of the main aims of this repository, known as SCAN (Schistosomiasis Collection at NHM), is to capture material now for future comparative studies. Samples of schistosomes or snails collected as part of control monitoring or research programmes often have a value beyond their immediate use. This is especially true when specimens are accompanied by the best possible acquisition data, so that the context of the specimens is well understood. Bringing good collections management practice to the fore when collecting in the field also has immediate benefits for project and data management. By providing a genetic snapshot of both parasites and snails fixed in time and space, SCAN will be providing a unique resource and research opportunity for current and future investigators.

#### NHM and molecular collections

NHM houses a collection comprised of some 70 million biological and geological specimens, including material originating from Sir Hans Sloane, Captain Cook, Charles Darwin, David Livingstone, Ernest Shackleton, and many others. The collection, including many type-specimens is an actively managed resource, is expanding, and is widely used as a source of reference and research material. Technological developments and the changing uses of the NHM collections have been recognized in NHM policy, allowing some controlled destructive sampling and development of collections specifically for molecular biological use. The centrepiece of this policy is the creation and construction of a molecular collections facility, MCF, completed in 2011. This facility is being used to store material suitable for molecular biological research generated from NHM’s own research projects, external collaborations and new collections.

SCAN is running within the MCF, whose collections are subject to the museum’s collection management policies and can be made available for third party research.

#### WWBL and SCORE

WWBL have a long history of working on schistosomiasis and are a designated WHO Collaborating Centre for the identification and characterization of schistosomes and their intermediate snail hosts. WWBL have maintained collections of schistosomes and snails including an archive of frozen schistosome tissue for many years. WWBL have existed in their current form since 2000 and were developed as purpose-built laboratory facilities for schistosomiasis research.

The existing WWBL collections of schistosomes and snail intermediate hosts are the result of many years of collection, research and maintenance by NHM researchers and international collaborators. The frozen collection of schistosomes is comprised mainly of vials containing 10–20 paired adult schistosome worms which have been acquired through passage of laboratory and field isolates since the 1980s and stored in liquid nitrogen. Many of the schistosomes have been collected from diverse regions of Africa and beyond (Table 1) and represent samples of 12 different species infecting humans and animals: * Schistosoma bovis,  S. curassoni *. * S. haematobium,  S. intercalatum,  S. guineensis,  S. japonicum,  S. mansoni,  S. margrebowei,  S. mattheei,  S. nasale,  S. rodhaini,  S. spindale *. WWBL have recently changed focus to direct sampling of schistosome miracidia and cercariae without the laboratory passage that requires animal use, and may distort population genetic inferences through population bottlenecks [6]. These new methods of field sampling and genotyping have resulted in these larval collections being stored in different media such as FTA cards (Whatman) or RNAlater (Ambion) [7,8]. Snail samples, primarily * Bulinus * spp. and * Biomphalaria * spp. are also held in ethanol, both purposely for DNA analysis, and also historically as in the case of an important and extensive collection of African freshwater snails compiled from 1947, which together with a dry shell collection were the working collections used by the late Dr Christopher Wright and Dr David Brown, authorities on African freshwater snails [9,10].

**Table 1:** Countries represented in the WWBL frozen sample collection

**Geographic area**	**Countries**
Mediterranean and Middle East	Spain, Egypt, Oman
Sub Saharan West Africa	Senegal, Gambia, Ginea Bissau, Liberia, Mali, Niger, Nigeria
Central, West Africa	Cameroon, DR Congo, Gabon, Zambia, Sao Tomé
East, Southern Africa	Sudan, Kenya, Uganda, Burundi, Tanzania, Zanzibar, Zimbabwe, South Africa
Indian Ocean, Asia	Sri Lanka, Mauritius, Madagascar, China
South America and Caribbean	Brazil, Guadeloupe

SCAN was set up in response to a specific need to retain specimens perceived by the steering group of a large, collaborative research programme, the Schistosomiasis Consortium for Operational Research and Evaluation (SCORE). SCORE is focused on * S.mansoni * and * S. haematobium *, and is concerned with operational research designed to enhance schistosomiasis control programmes and evaluate an effort to eliminate this parasite from a large geographic area (http://score.uga.edu). Specifically the activities of SCORE are based around 3 objectives:

· Objective 1: Evaluate alternative approaches to control schistosomiasis, and to eliminate schistosomiasis where possible

· Objective 2: Develop the tools needed for a global effort to control and eliminate schistosomiasis

· Objective 3: Assist in maximizing the global schistosomiasis control and elimination effort and its integration into broad-based NTD control programmes.

Sampling is being undertaken as part of the programme and these studies are providing the initial source of material of SCAN.

### Aims

SCAN aims to provide facilities that will allow deposition and retrieval of schistosomiasis-related specimens in a managed collections infrastructure. The long-term purpose of the facility is to provide geographic and temporally distinct samples for retrospective, comparative genetic/genomic analysis. The intention is that the interests of the collector and potential users should be balanced, and projects are undertaken in a spirit of collaboration. In addition to the samples themselves, SCAN aims to maintain high quality accompanying data to provide the necessary context and maximise research value of the specimens.

#### WWBL collections and integration with MCF

Existing WWBL specimens will be incorporated into SCAN and made available for approved research use, thereby securing long-term maintenance within a world-class collections management infrastructure.

#### SCORE programme

Representing the interests of a large section of the schistosomiasis control and research communities, SCORE has identified the need for a repository to support its sampling projects and the SCORE secretariat and advisory committee provided the impetus to seek funds for a repository. SCAN has already incorporated samples from baseline sampling projects within the SCORE programme, and this work will continue throughout the 4 year study period.

#### Other research

SCAN is available both to provide and receive specimens for research purposes. Submission of samples to SCAN opens possibilities for future use, thereby increasing the benefits of the sampling programme. Holding the samples in a central repository will allow consistency of data quality, storage conditions and curation. Access to the specimens can be granted upon successful application through NHM collections management procedures (see below).

The next decade will see an increased push not only to control morbidity caused by schistosomiasis but also more intense efforts to eliminate this disease in certain settings [11]. Recent announcements of increased drug donations and funds for implementation suggest that the disease landscape is set to change and it would be timely to store representative parasite samples as part of monitoring and surveillance.

### Policies and procedures

**Figure 1:**
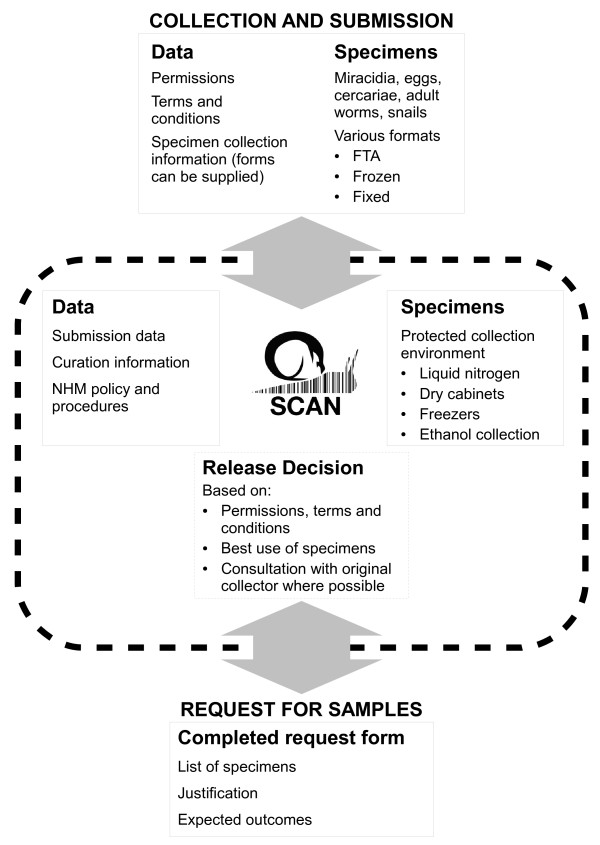
Summary of SCAN operating procedures.

SCAN procedures are summarised in Figure 1.

#### Submissions

NHM is governed by specific UK legislation that dictates the terms upon which it can accept specimens [12,13]. International agreements such as the Convention on Biological Diversity (CBD) also inform NHM policy and affect the regulatory frameworks within which the museum operates [14]. While specific terms and conditions may be incorporated into collections agreements, the general policy is for NHM to seek title to the specimens it holds, thus safeguarding specimens by allowing NHM to take full responsibility for them. Items to which NHM does not hold title are treated as long-term loans, which might necessitate return to country of origin, with associated open-ended expense or sample destruction. Specimens submitted to SCAN will therefore require appropriate title/material transfer agreements, but unless stated otherwise the principle of NHM title should be assumed with any material submission. The interests of the collector will be taken into account in providing access and when considering third-party use, with specific requirements embedded in the transfer agreements. Ethical statements and collection permission documents will also be required as appropriate.

#### Legacy

Documentation and comprehensive data for some of the “legacy” material maintained in WWBL’s research collections, which will be transferred to the repository may not be available. For these specimens the authors can make the assurance here that specimens acquired by WWBL have been collected in collaboration with local authorities abiding by the ethical standards and collection requirements of the day. Laboratory animal use has been within a designated facility regulated under the terms of the Animals (Scientific Procedures) Act, 1986.

#### Storage methods

Existing WWBL collections include adult schistosomes frozen in liquid nitrogen, larval schistosomes stored on Whatman FTA cards, and snails stored in ethanol, and SCAN is open to negotiation regarding the storage media used for specimen submission. Although WWBL has maintained and still maintains live material in the laboratory, the repository itself will not undertake laboratory passage or provide live material. Live material is available from other sources, such as the NIAID Schistosomiasis Resource Center [15].

As a general rule, we wish to encourage direct sampling of schistosome larval stages in field situations. One possible drawback of this policy is that DNA quantity will be severely limited. To maximize use of samples, we also encourage the use of whole genome amplification techniques where possible. Such techniques have been successfully used to provide material for microsatellite genotyping, which is one of the more challenging uses of this technology [16].

#### Access and benefit sharing

Requests for access to the collection will be assessed according to NHM collection management policies. Specimens will be released based on the merits of the research intended, the capacity of the researcher to carry out the work and the value of the specimens. The original terms and conditions of the collection agreements (including access embargoes) will be taken into account, and collaboration between the original collecting team and the party requesting access will be encouraged.

While the CBD has been effective in promoting conservation, concerns have been expressed that a combination of the fear of biopiracy among developing countries and fear of legal hurdles among the developed has inhibited research partnerships [17]. SCAN aims to stimulate research partnerships by providing benefits to the research community such as follows:

· Widening access to schistosomiasis research samples;

· Facilitating follow-up studies as patterns emerge or techniques change;

· Maintaining a snapshot of genetic diversity of populations that may be eliminated or heavily reduced;

· Maintaining specimens associated with particular datasets to allow repeat analysis and independent verification.

**Figure 2:**
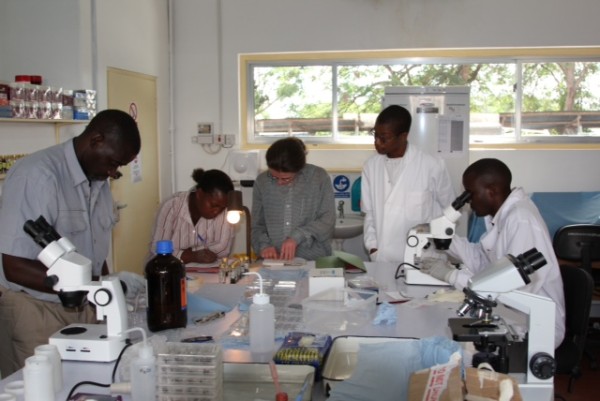
**Collaborative work with SCORE project team at National Institute for Medical Research (NIMR), Mwanza, Tanzania.** From left, James Kubeja (NIMR), Teckla Angelo (NIMR), Fiona Allan (SCAN), Safari Kinung’hi (NIMR), Emmanuel Boniphace (NIMR).

Encouraging collaborative research brings the potential of further benefits such as assistance with collection activities, training and capacity building (Figure 2). WWBL have a track-record of such collaborations, and welcome new initiatives.

Requests for use of specimens should be made to SCAN (a.emery@nhm.ac.uk). In order to gain access, the potential user will be required to provide information on the use to which the specimens will be put, and provide evidence of their capacity to carry out the work. Requests will be considered, as elsewhere in NHM, by the local curation team and departmental head of collections. The interests of the collector, user, NHM and wider community will be taken into account. Users should gain access agreement at an early stage, and certainly prior to funding applications for work dependent on access. SCAN will aim to make a decision within two weeks of application.

#### Cost recovery

SCAN is supported with funding from the Wellcome Trust and NHM Grant-in-Aid. Concentration on low-maintenance curation methods and working within NHM collection management will ensure that SCAN specimens can be maintained. However, as resources are limited, further collection enhancement by developing new partnerships, and projects using specimens will require separate funding. Where possible, this would be through collaborative partnership.

### Progress and vision

Prior to setting up SCAN as a formal repository, specimens retained in the WWBL collections have been used for a range of projects including population genetic marker development, DNA diagnostic tools, DNA barcoding and evolutionary/phylogenetic analysis [10,18-21]. The collection has also provided material for retrospective analysis, for example after schistosome hybridization emerged in Cameroon and DNA-based techniques to identify hybrid genotypes emerged [22]. Since the project started, SCAN working in partnership with the NHM’s molecular collection facility, has set up the core facilities necessary for sample storage. Working with the SCORE baseline survey teams, SCAN has acquired in excess of 45,000 larval schistosome samples from Tanzania, Zanzibar and Niger. Some of these samples will be used to complete population genetic surveys and the rest will be retained for future work.

SCAN is a forward thinking NHM based programme that is collecting and storing samples today that may be of interest to research scientists in the future. In order for SCAN to grow and be successful, we invite all those currently involved in schistosomiasis field research or control programmes to consider submitting samples to the collection. Both schistosome and snail populations are under increasing selection pressures, be they man made or environmentally induced, samples gathered now will provide a series of genetic snapshots for future reference and comparative studies.

## Consent

Written informed consent was obtained for publication of the images in this report.

## Competing interests

AE has received funding for a separate project from GE Healthcare, manufacturer of Whatman FTA cards.

## Authors’ contributions

AE drafted the manuscript and manages the SCAN project. FA and MR developed repository and data-handling systems, coordinated fieldwork and provided manuscript edits. DR revised the manuscript and coordinated fieldwork and links between SCAN and SCORE. All authors read and approved the final manuscript.
